# Tips and tricks in the dermoscopy of pigmented lesions

**DOI:** 10.1186/1471-5945-12-14

**Published:** 2012-08-24

**Authors:** Grazyna Kaminska-Winciorek, Radoslaw Spiewak

**Affiliations:** 1 Department of Experimental Dermatology and Cosmetology, Faculty of Pharmacy, Jagiellonian University Medical College, ul. Medyczna 9, 30-688, Krakow, Poland

## Abstract

Dermoscopy is a useful, widely used tool for examining pigmented lesions, especially helpful in cases of an uncertain nature. Nevertheless, doctors may experience diagnostic difficulties while using this method. An example of this may be found in the examination of subcorneal hematoma, dark nevi with black lamella or lesions of acral volar skin. In such cases, a few diagnostic tricks have proven to be helpful in achieving diagnostic accuracy.

This paper reviews various methods of performing dermoscopy, suggesting a number of simple, yet helpful tests. These include the adhesive tape test, the skin scraping test and the ink furrow test. The adhesive tape test is helpful in differentiating between dark melanocytic nevi and melanoma. Hematoma may be more easily differentiated with the use of the so-called skin scraping test. The confirmation of benign and melanocytic lesions of acral volar skin, on the other hand, is more accurate when using the ink furrow test. These methods have been discussed here based upon a series of literature reviews, the authors’ own experience and, also, iconography.

The present article describes novel methods used in dermoscopy, helping to bring about a faster, more accurate diagnostics of those lesions which have proven to be more difficult to recognize. Helpful tricks, such as have been known to professional literature, as well as the authors’ own experience (for instance, applying urea cream to hyperkeratotic lesions or using photographs of skin lesions taken with the aid of a mobile phone camera – all prior to surgery) will surely be considered beneficial to the practitioner, be it dermatologist or any other physician.

## Background

Dermoscopy (synonyms include terms such as epiluminescence microscopy, skin surface microscopy, incident-light microscopy) has, of late, made a name for itself as a greatly appreciated method of dermatological diagnosis. Initially intended for the differential diagnosis of pigmented lesions, dermoscopy became more widespread in the 1990s. To the present day, dermoscopy is used in assessing inflammatory dermatoses (inflammoscopy), parasitic invasions (the so-called “entomodermoscopic method”) and in cases of scalp disorders (trichoscopy) - all in the follow-up to dermatological treatment.

Performing dermoscopy during dermatological examination should really be adapted as a routine activity. Although a complete and thorough examination of the skin (CSE) with the use of a dermoscope is, effectively, more time-consuming, it is strongly advisable to dedicate the incurring three or four additional minutes (compared to a traditional dermatological examination without the use of dermoscopy) to increase the detection sensitivity of potentially fatal skin malignancies [1]. Dermoscopy may be carried out with the aid of classic dermoscopes, stereomicroscopes, dermoscopes connected to a digital camera, or even videodermoscopes - in which an image obtained through a video camera is sent to a computer screen. At present, technologically advanced, high resolution digital cameras, built into videodermoscopes, considerably improve image resolution and enhance the quality of the obtained images. Moreover, the development of special dermoscopic extensions allows for the conversion of top-end mobile phones into pocket dermoscopes. This provides an opportunity for a quick analysis of the image, transferring the photo via MMS and email and thus creating a type of ‘handheld’ patient database.

## Methods

The article describes a number of practical dermoscopic methods which can be implemented in order to achieve a faster and more accurate diagnostics of lesions which have proven to be difficult to recognize. Professional literature has played host to many of these over the years, yet the authors have no doubt that the methods described here (all based on their own medical experience) shall be appreciated by dermatologists and general practitioners alike. We have described these useful tricks and tips based on literature review and our own work with dermoscopic figures. Written consent has also been obtained from the patients for the publication of the accompanying data as well as any associated images. A copy of this written consent is available for review at the discretion of this journal’s Series Editor.

## Results and discussion

### Examination techniques

#### Types of dermoscopic devices

The majority of doctors who perform dermoscopy begin their evaluation of melanocytic nevi with manual dermoscopes, which allows for the direct visual inspection of the lesion and helps in avoiding possible distortions of colour, structures and edges of the lesion in question – all while transmitting this data to a computer. Research conducted by Seidenari et al. [2] confirmed that the type of equipment used does not influence the identification of specific dermoscopic features. However, smaller magnifications used for archiving the lesions may decrease the quality of images [2].

#### Immersion in non-polarized light or evaluation in polarized light?

**Figure 1:**
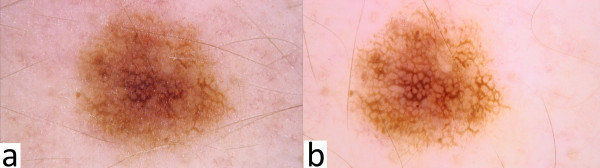
ab. Dermoscopic image of melanocytic nevus in non-polarized light source without immersion (Figure 1a) compared to non-polarized light source with immersion (Figure 1b).

Immersion is routinely used for the dermoscopic evaluation of melanocytic nevi in order to increase the translucency of stratum corneum and the visualization of dermoscopic structures (Figure 1a and b). It reduces the reflection of light from the skin’s surface and enhances the transparency of the stratum corneum. The result is that the visibility of pigment-containing structures in the epidermis is largely enhanced. This is also true for the clarity of the image of the dermo-epidermal junction and the upper dermis [3]. The most common immersion fluids in dermoscopy are synthetic oil, ultrasonographic gel, alcoholic disinfectants or, simply - water. Ultrasonographic gel seems to be the best immersion fluid - it is inexpensive, efficient, and ensures a good adhesion of the dermoscope to the lesion - making it possible to analyse not only flat melanocytic nevi but also those of verrucous type, possessing crypt-like depressions. When using USG gel, less pressure is required to obtain a better visibility of the vascular structures within the lesion. Furthermore, the gel facilitates dermoscopic revision in difficult-to-access regions such as the flexures of extremities and interdigital spaces. Ultrasonographic gel assures appropriate immersion while avoiding any staining on the dermoscope’s optical systems (as well as patients’ garments) while being rather inexpensive [4]. ECG gel should be avoided, as it may leave permanent stains on the dermoscope’s optical system, especially on its rubber elements.

Nowadays, polarized light dermoscopes in which immersion is not required are becoming more and more popular. Dermoscopic structures such as milia-like pseudocysts, comedo-like openings, bluish-grey homogenous areas, peppering-like, white-bluish structures with regression and brightly-coloured areas - all these can now be better visualized as compared to detection and revision with the use of non-polarized dermoscopes. The latter ones, it must however be said, are effectively better in assessing vascular structures and red areas. Non-polarized dermatoscopes are also more effective in showing pigmented lesions (increased melanin deposition), and blue nevi [4,5]. In order to combine the benefits of both types, dermoscopes with dual light-sources (non-polarized and polarized) have now been designed. In some cases, the dermoscope is used without immersion (so-called dry dermoscopy), for example in assessing the skin of the scalp, vellus or terminal hair, or in the case of examining dermatoglyphs or sweat glands in the palms and feet [6].

#### Inspection of all lesions or only those “ugly ducklings”?

A common mistake is to limit dermoscopic assessment only to lesions clinically suspicious or pointed out by a patient as an “ugly duckling”. Although the “ugly duckling” is usually a sign typical of melanoma [7], all existing melanocytic lesions must undergo dermoscopic evaluation, regardless of their location and size. According to Bono et al. melanomas are more frequently diagnosed among minor melanocytic lesions (less than 3 mm in size) than among those of greater diameter, which usually spark more concern among patients [8,9]. So-called “micromelanomas” in the form of foci measuring less than 3 mm in diameter constituted 2.4% of all examined melanomas (22 of 924 cases of primary foci of melanoma) [8]. In a further study using the same test group [9], melanoma was diagnosed in as many as 23 of 206 pigmented lesions of a diameter less than 3 mm. Compared to a clinical examination of pigmented lesions smaller than 3 mm in diameter (sensitivity for the detection of melanoma 43%, specificity 91%), dermoscopy shows a much higher sensitivity for the diagnosis of melanoma (83%) and lower specificity (69%) than clinical examination.

The classification of atypical nevi proposed in 2009 by Marghoob et al. [10] includes patterns of melanocytic nevi of the “Beauty and the Beast” type, emphasizing the assessment of overall symmetry of colour and form as a means of distinguishing benign (also dysplastic) nevi from melanoma [10]. Melanomas can display one of the “Beast” structures such as anatypical network, globules and dots, streaks (radial streaming or pseudopods), eccentric blotches, a blue-white veil, a negative pigmented-network, regression structures and abnormal vascular formations. Among atypical nevi of the “Beauty” type, cases of the so-called “wolf in sheep’s clothing” have been observed, where a seemingly benign nevus in fact proves to be a melanoma [10].

The “4 × 4 × 6 rule” postulated by Zalaudek et al. [11] is meant to support the clinicians’ memorisation of basic dermoscopic patterns of nevi, along with all other factors influencing specific dermoscopic patterns. Four dermoscopic criteria affect the evaluation of melanocytic nevi. These are: colour (black, brown, blue, grey), pattern (globular, reticular, starburst and homogeneous blue), distribution of pigment (multifocal, central, excentric and uniform) and specific location (face, acral volar skin, nail plate, mucous membrane) and 6 factors affecting the dermoscopic pattern (age, skin phototype, medical history of melanoma, UV exposure, pregnancy and dynamics of growth). It is not only the dermoscopic pattern, but also the circumstances and factors influencing its formation and evolution that determine proper therapeutic management. These factors have been discussed in detail in a recent paper by Zalaudek et al. [11]. In the dermoscopic assessment of melanocytic nevi, the dermoscopic pattern is often related to the patient’s age, as well as the location and duration of the nevus. Among children younger than 11 years of age, the globular pattern is most common, especially on the trunk, while the reticular pattern can more often than not be found on the extremities [2]. In the case of patients above 15 years of age, reticular and homogeneous patterns prevail [12]. Thus, the reticular pattern, typical for junctional nevi usually induced by exogenous factors such as exposure to UV radiation [13], is more often observed on sun-exposed areas.

**Figure 2:**
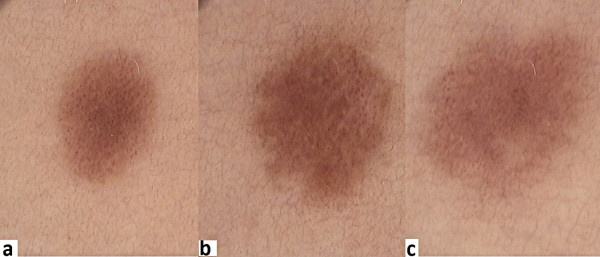
**abc. A profile of melanocytic nevi: right thigh, woman, age 30, skin phototype I, red hair.** Dermoscopy reveals light-brown colour with shades of pink, central hypopigmentation and numerous dotted-type vessels.

In the assessment of patients with melanocytic nevi, consideration of the patient’s “nevus type” may be helpful [14]. This “nevus type” refers to the typical (prevailing) pattern of the patient’s nevi. More suspicion should be directed against nevi not consistent with the prevailing pattern of nevi (“nevus type” of the patient). Only the evaluation of all nevi allows us to define the “nevus type” of the patient examined [14]. A nevus may often look more suspicious among people with a very fair phototype, when a light-rose colouring is observed with a central hypopigmentation and numerous vessels, frequently resembling dots. However, after finding more nevi following the same initially suspicious dermoscopic patterns, the examiner becomes reassured, because in fact it reflects the patient’s typical “nevus type” (Figure 2a, b and c). As Zalaudek et al. [15] have demonstrated, while the above described pattern is more frequently observed among people with fair skin phototype (Fitzpatrick phototype I), central hyperpigmentation and nevi of dark-brown colouring are more prevalent in people with darker skin (phototype IV). And while the reticular pattern is more common among people with darker skin complexion, it is also dominant in adults with all skin phototypes [15].

### Additional dermoscopic tests

#### The adhesive tape test

##### The “ugly duckling sign” and black lamella

**Figure 3:**
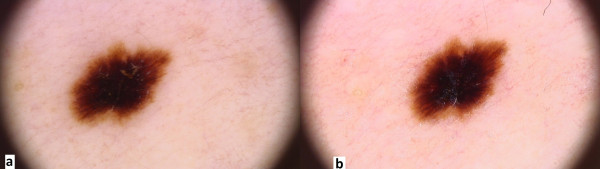
**a. Dermoscopic image of melanocytic nevus prior to adhesive tape test. b.** Dermoscopic image of melanocytic nevus following adhesive tape test (pigment network is more visible, black lamella has been torn off).

Doctors are frequently concerned about the presence of dark (brown or black) homogeneous areas involving the central part of a nevus called “black lamella”, characteristic of the so-called dark nevi (Figure 3a). This lamella corresponds histopathologically to a cornified layer containing large amounts of melanin granules and, after careful removal, a reticular area typical of the acquired nevi becomes more visible. The adhesive tape test facilitated in the diagnostic process is to confirm that the “ugly duckling sign” is only temporary. The test involves the repeated sticking of adhesive tape to the lesion, and then tearing it gently off, which results in the removal of the central hyperkeratotic black lamella [15] (Figure 3b). We suggest that, in case of verrucous nevi with crypt-like depressions filled with yellowish hyperkeratotic masses, a 5% urea cream be applied to the lesions for several days before dermoscopy – this in order to dissolve the keratin mass.

#### The ink furrow test

##### Diagnosis of acrally located pigmented lesions

**Figure 4:**
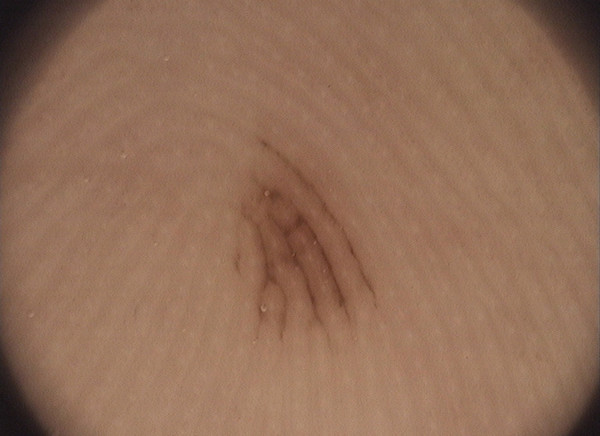
Dermoscopic image of acral melanocytic nevus with typical parallel furrow pattern.

Melanocytic lesions of the hands and soles may be divided into four major patterns, which are associated with the characteristic distribution of melanocytes, reflecting the anatomical structure of the skin in these body parts [16-18]:

– The parallel furrow pattern: a darker colour runs along the sulci; this is the most common pattern of acrally located benign melanocytic lesions (Figure 4),

– The fibrillar pattern: the pigment is distributed along numerous fine lines which cross the sulci

– The lattice-like pattern: this is characterized by the presence of bands of pigment, distributed parallel and transverse to the sulci

– The non-typical pattern: one in which no characteristic features can be defined; this type however, is not equivalent to the so-called multicomponent pattern

Melanocytic nevi showing the parallel furrow or fibrillar pattern are predominantly located on the foot, in which case a regular parallel distribution of skin markings is observed. However, as a rule, these nevi rarely affect the arch of the foot [19]. Additionally, the fibrillar pattern of melanocytic nevi indicates a predilection for the load bearing areas of the foot [19]. The lattice-like pattern is mainly found on the arch area of the foot [19].

Other patterns have also been recognized, among them the parallel ridge pattern – in which the pigment is arranged along the crista, as opposed to the parallel furrow pattern. The parallel ridge pattern is mainly to be found in melanoma *in situ* or in the early stages of invasive acral volar skin melanoma. The parallel ridge pattern shows a high specificity (99%) in the detection of melanoma of acral volar skin, especially in its early stages [16]. Among less common patterns, there is also the globular pattern – in which pigmented globules present a regular distribution inside the nevus. Apart from this, there is also the homogeneous pattern in which a homogeneous area of light-brown to dark-brown colour is predominant with blue pigmentation, and the reticular pattern, which is similar to a basic reticular pattern typical of melanocytic nevi and ranging from light-brown to dark-brown colour. The transitional pattern combines a brown to dark-brown pigment network with a parallel orientation of pigment bands. In clinical trials, the most common pattern of acral melanocytic nevi was the parallel pattern (42-50%), lattice-like pattern (12-15%), the reticular pattern (15-20%) and atypical pattern (ranging from 10-13% up to 65% of all investigated melanocytic nevi) [17,18]. Other patterns of melanocytic nevi observed included the fibrillar (10-38%), homogeneous (9-13%), globular (5-20%) and reticular pattern (2-40%) [17,18]. The transitional pattern had been noted in 1.8% of cases [17].

**Figure 5:**
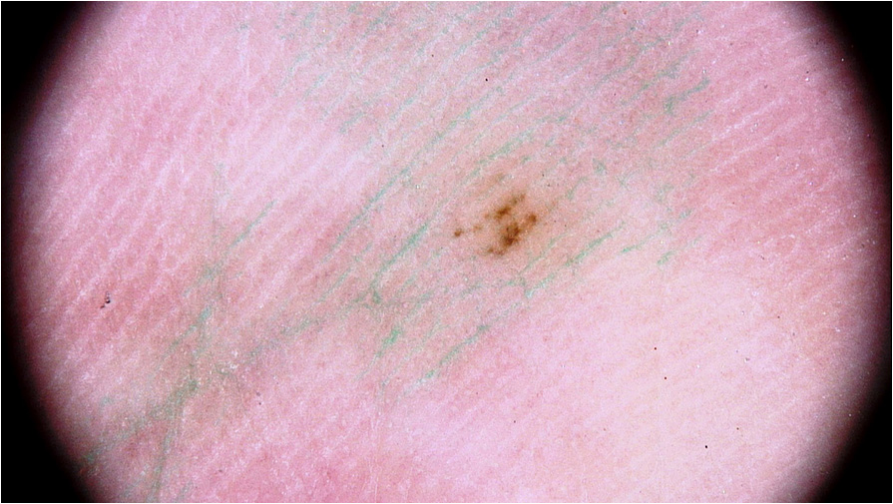
Ink furrow test: the ink settles in the sulcus, which correlates with the dispersion of the dye in a mild case of melanoma.

The pigment of the acral nevi is mainly located within the furrows of skin markings (the parallel furrow pattern), whereas melanoma shows pigmentation on the ridges (the parallel ridge pattern) [16,20]. Sometimes, it is not easy to distinguish the location of the pigment, whether it concerns either furrows or ridges, and this makes the diagnosis of a pigmented lesion much more difficult. In doubtful cases, Braun et al. [21] recommend a simple ink furrow test. This in turn will make it easy to evaluate whether the melanin pigmentation follows the ink lines (the benign pattern) (Figure 5) or if the pigmentation is located between these ink lines (the malignant pattern). The ink furrow test is both simple to apply and readily available. Liquid ink (e.g. from a fountain pen) should be applied onto the skin and left for a few seconds to allow the ink to penetrate the furrows. The excess ink should be wiped off using a cotton swab. The subsequent cotton swab wiping will only remove the ink from the skin overlying the ridges. The furrows will retain the stain and become clearly visible for dermoscopic examination in the form of thin, ink-stained lines [20]. According to Uhara et al. [22], staining with liquid ink from a fountain pen sometimes shows a fuzzy image because the ink can be easily removed from the furrows by wiping. Therefore, the authors propose using whiteboard marker pens containing alcohol-based ink [22].

#### The scraping test in diagnosing lesions of parallel ridge pattern

##### Hematoma or melanoma?

**Figure 6:**
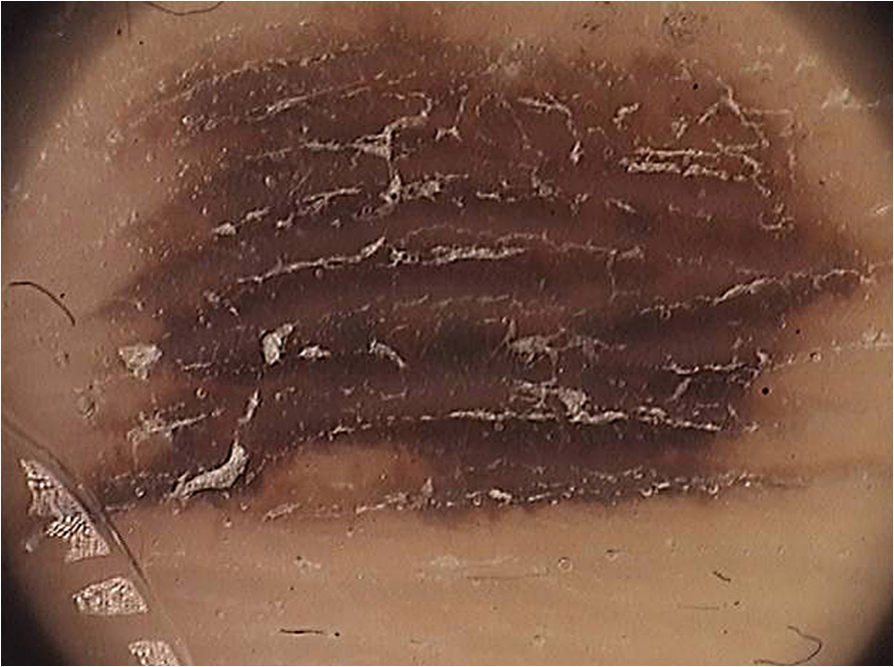
Dermoscopic image of subcorneal hematoma of the feet shows typical parallel ridge pattern.

**Figure 7:**
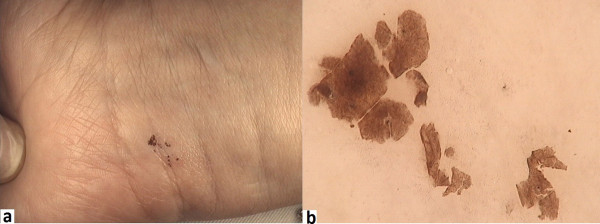
**a. Macroscopic image of subcorneal hematoma. b.** Dermoscopic image of hematoma after scraping test- homogenous brownish masses are visible.

The dermoscopic pattern of subcorneal hematomas bears a stark similarity to melanoma. The parallel ridge pattern is to be observed in up to 40% of hematomas [23] (Figure 6). Ishihara et al. [24] suggests that this parallel ridge pattern - a band-like pigmentation on the ridges of the skin markings, is highly specific to melanoma *in situ* on acral volar skin (on the palms and soles). In this study, the authors examined 22 acral melanocytic lesions which showed the parallel ridge pattern in dermoscopy - 20 of these were diagnosed as early melanoma *in situ*[24]. A dermoscopic examination of hematomas usually reveals reddish-black homogeneous areas, often accompanied by satellite globules [24]. In doubtful cases, a scraping test is pivotal for the confirmation of hematoma. The test is easy to perform and involves the gentle scraping off of the stratum corneum of the epidermis with a sterile scalpel or needle, which results in a complete or partial removal of the pigmentation in the parallel ridge pattern – a feature characteristic for vascular lesions, which is not observed in pigmented ones [24]. Figure 7 presents a typical subcorneal hematoma, which partially disappeared after the scraping test.

## Conclusions

**Figure 8:**
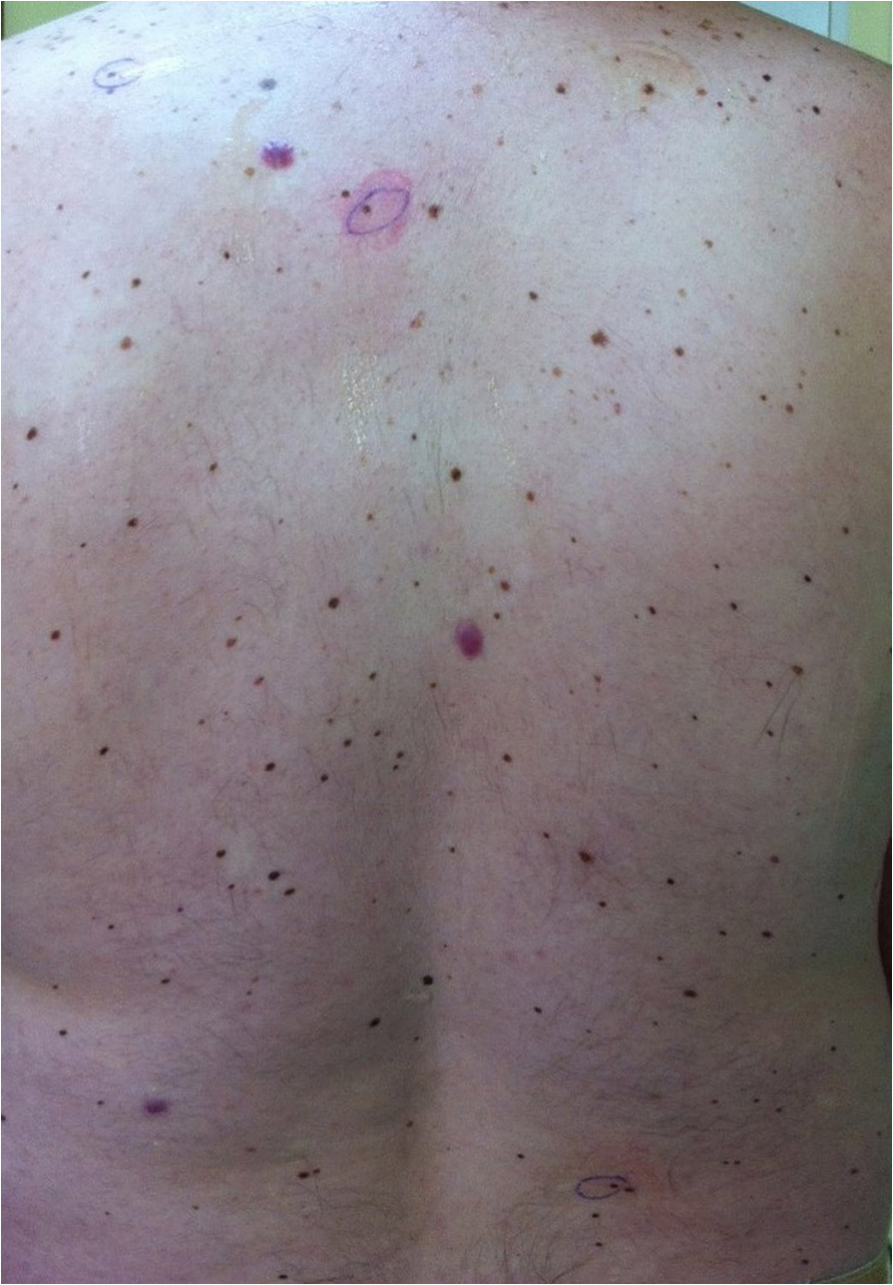
**Marking of suspected lesions before surgical removing.** The patient can easily point the suspected skin lesions after performed dermoscopy, especially in the atypical mole syndrome - while above 300 lesions can exist on whole the body.

This paper collates simple dermoscopic tests which can be carried out in cases of diagnostic difficulties. The tests are simple, fast, safe and cost-effective, and they may be especially helpful in doubtful cases or in situations when the examiner only has a basic knowledge of dermoscopy. The authors also suggest taking a photograph (with the use of a mobile phone camera, for instance) of suspicious skin lesions which will to be excised for easy marking and identification of the lesions during conducted surgery (Figure 8).

## Competing interests

The authors declare that they have no competing interests.

## Pre-publication history

The pre-publication history for this paper can be accessed here:

http://www.biomedcentral.com/1471-5945/12/14/prepub
